# Comparative Effects of Behavioral Analysis Therapy of Feelings and Brief Focal Psychotherapy: Protocol for a Randomized Controlled Assessor-Blinded Clinical Trial

**DOI:** 10.3390/medsci14050551

**Published:** 2026-09-08

**Authors:** Manoel Victor Fernandes Marques, Emerson Arcoverde Nunes, Rodrigo Costa de Oliveira, Nicole Leite Galvão Coelho, Glauco Francisco Silva, Mariana Dantas de Carvalho Vilar, Grasiela Piuvezam

**Affiliations:** 1 2nd Floor, Center for Health Sciences, Health Campus, Postgraduate Program in Health Sciences, Federal University of Rio Grande do Norte (UFRN), Av. Gal. Gustavo Cordeiro de Farias, s/n, Natal 59012-570, RN, Brazil; manoelvicfm@gmail.com (M.V.F.M.); marivilarnutri@gmail.com (M.D.d.C.V.); 2 Systematic Review and Meta-Analysis Laboratory (UFRN-CNPq), Postgraduate Program in Public Health, Campus Lagoa Nova, Federal University of Rio Grande do Norte (UFRN), Natal 59078-970, RN, Brazil; 3 Department of Clinical Medicine, Hospital Universitário Onofre Lopes (HUOL), Federal University of Rio Grande do Norte (UFRN), Brazilian Hospital Services Company (EBSERH), Av. Nilo Peçanha, 620, Petrópolis, Natal 59012-300, RN, Brazil; emerson_arcoverde@yahoo.com.br (E.A.N.); rodrigo.coliveira@ebserh.gov.br (R.C.d.O.); 4 Department of Physiology and Behavior, Center of Biosciences, Federal University of Rio Grande do Norte (UFRN), Av. Sen. Salgado Filho, 3000, Lagoa Nova, Natal 59064-741, RN, Brazil; nicole.galvao@ufrn.br; 5 Clinical Research Center, Hospital Universitário Onofre Lopes (HUOL), Federal University of Rio Grande do Norte (UFRN), Brazilian Hospital Services Company (EBSERH), Av. Nilo Peçanha, 620, Petrópolis, Natal 59012-300, RN, Brazil; glaucoestatistico@gmail.com

**Keywords:** anxiety, depression, psychotherapy, biomarkers, randomized controlled trial

## Abstract

Background: Brief psychotherapies have been shown to improve clinical symptoms within a limited number of sessions without compromising treatment quality. Objectives: This study aims to test the effects of the experimental Behavioral Analysis Therapy of Feelings (BATF) in a randomized controlled trial design. Methods: This randomized, controlled, assessor-blinded clinical trial compares BATF with Brief Focal Psychotherapy in individuals presenting symptoms of anxiety and/or depression. Participants with substance use disorders, tobacco or alcohol use, pregnancy, or use of oral contraceptives will be excluded. Participants will be randomized in a 1:1 ratio with stratification by clinical condition. Outcomes will be assessed at baseline, Week 4, and Week 13 using the Hamilton Anxiety Rating Scale, the Montgomery–Åsberg Depression Rating Scale, the Patient Health Questionnaire-9, the Generalized Anxiety Disorder-7, and the Pittsburgh Sleep Quality Index. In addition to clinical outcomes, exploratory salivary and blood biomarkers associated with stress, inflammation, and neurobiological function will be evaluated. The trial is registered in the Brazilian Clinical Trials Registry (RBR-10p45rdg) and has Universal Trial Number U1111-1313-4499. Conclusions: This protocol describes a randomized controlled trial comparing BATF with Brief Focal Psychotherapy. The study is designed to generate preliminary evidence on the effects of the intervention in anxiety and depressive symptoms.

## 1. Introduction

Anxiety is linked to impairments in psychosocial well-being, quality of life, and the perception of negative effects associated with depressive symptoms [1]. Mild to moderate anxiety and depressive symptoms may benefit from brief psychotherapeutic interventions, which have demonstrated effectiveness in reducing emotional distress [2]. Treatment selection should be individualized according to symptom severity, diagnostic characteristics, and clinical context, and may include psychotherapy, pharmacotherapy, or their combination when clinically indicated. These interventions are characterized by a limited number of sessions, a focus on the patient’s primary complaint, and clearly defined objectives aimed at achieving improvement within a short period of time. Examples of brief psychotherapeutic approaches include Brief Focal Psychotherapy [3]. Both brief and conventional psychotherapies demonstrate favorable immediate responses and maintain effectiveness over extended follow-up periods [4,5].

A limitation that has been described in structured models of brief psychotherapy is the reduced capacity to produce broader modifications in core psychological functioning, mainly due to the emphasis on the primary presenting complaint, the restricted duration of treatment, and the time required for participants to develop new skills capable of influencing deeper emotional and behavioral patterns [6,7].

The BATF was developed with the aim of promoting broader changes in emotional, cognitive, and behavioral functioning; however, these effects remain hypothetical and will be empirically evaluated in the present randomized controlled trial. In response to this gap, the present study proposes to evaluate a brief psychotherapeutic intervention that targets the primary complaint and is hypothesized to produce broader functional changes compared to an established brief psychotherapy. This study may contribute to the development of brief psychotherapeutic approaches.

BATF is a manualized and structured brief psychotherapeutic intervention currently under empirical evaluation. BATF is proposed to differ from existing brief psychotherapies by its procedural architecture, which operationalizes affect as a structured input for behavioral planning through a fixed sequential system of affective identification, thematic consolidation, and behavioral translation. Unlike traditional brief psychotherapies focused on insight generation or focal conflict exploration, BATF emphasizes the systematic conversion of affective states into actionable behavioral outputs within a standardized protocol. The procedural sequence is identical across participants, with variability arising only from the individualized emotional content and behavioral goals generated within the protocol-guided framework.

BATF is based on the assumption that clinically relevant affective experiences may not be immediately accessible through reflective cognitive processing alone. The intervention proposes that psychological distress may be partially maintained by difficulties in identifying, organizing, and behaviorally integrating pre-reflective affective experiences. Rather than initially emphasizing interpretation or cognitive restructuring, BATF employs a structured sequence designed to facilitate direct observation of affective experiences in their immediate manifestation. Through sequential affective identification, thematic organization, and behavioral translation, the intervention seeks to increase the cognitive accessibility and functional integration of affective information. This process is hypothesized to facilitate behavioral planning, adaptive decision-making, and reduce maladaptive emotional reactivity. The central hypothesis underlying BATF is that the systematic organization of affective experiences increases their cognitive accessibility and behavioral integration, thereby facilitating adaptive action and reducing symptoms of anxiety and depression.

BATF was developed as a novel brief psychotherapeutic intervention and was not derived from Brief Focal Psychotherapy or any other established psychotherapeutic model. The intervention employs a structured sequence of affective identification, thematic organization, and behavioral translation designed to facilitate adaptive behavioral planning. Although BATF was not developed within any single traditional psychotherapeutic school, its procedural structure was informed by a phenomenologically oriented focus on the description of affective experience as immediately lived [8] and by the hypothesis that difficulties in identifying, organizing, and behaviorally integrating affective experiences may contribute to psychological distress. These principles were subsequently operationalized in BATF through a standardized sequence of affective identification, thematic organization, and behavioral translation designed for empirical evaluation. This rationale is broadly consistent with conceptualizations of emotion regulation that emphasize awareness, understanding, and behavioral integration of emotional experiences as relevant components of adaptive psychological functioning [9]. Its core procedural structure is described in the Supplementary Materials S1.1 to ensure reproducibility, treatment standardization, and protocol transparency. A more extensive technical and theoretical manual has been developed for therapist training purposes and will be reported in future publications following completion of clinical validation. To date, BATF has not been evaluated in randomized controlled trials. The current trial evaluates BATF in participants with anxiety and depressive symptoms, as these conditions were selected as the initial target population for empirical investigation. However, this study population does not define the theoretical or clinical scope of the intervention, which remains open to future empirical evaluation across other conditions. Additionally, the intervention has been designed to require fewer sessions than traditional brief psychotherapies, and its effects remain to be empirically tested.

The comparative analysis of the two brief psychotherapies in this study will be conducted using validated clinical questionnaires. Biological biomarkers will be collected as exploratory outcomes. This approach is supported by previous studies demonstrating that important advances in psychotherapy research involve combining clinical assessment instruments with biomarker evaluation. Effective treatments are expected not only to reduce symptoms but also to demonstrate measurable clinical significance [10].

Accordingly, exploratory biomarkers associated with stress regulation, immune-inflammatory activity, and neuroplasticity were included to complement clinical outcome assessment. These biomarkers were selected because previous studies have linked them to biological processes involved in anxiety and depressive symptoms, including hypothalamic–pituitary–adrenal axis functioning, inflammatory signaling, and neurobiological adaptation to psychological interventions.

To further characterize treatment effects, biomarkers may reveal dynamic molecular alterations associated with disease processes and clinical improvement; in this context, biomarkers such as cortisol may reflect normal biological processes, pathogenic alterations, or responses to therapeutic interventions [11] and are increasingly incorporated into clinical psychological research [12]. They are commonly used to evaluate treatment outcomes and monitor symptom progression alongside clinical rating scales [13].

In view of the foregoing, this study reports the protocol of a randomized controlled trial evaluating BATF, a novel brief psychotherapeutic intervention. BATF will be compared with Brief Focal Psychotherapy, an established brief psychotherapeutic approach widely used in clinical practice. The trial aims to evaluate the effects of BATF on symptoms of anxiety and depression and to explore associated clinical and biomarker outcomes.

## 2. Methods and Analysis

### 2.1. Trial Design and Registration

This protocol was registered on 6 November 2024, in the Brazilian Clinical Trials Registry (ReBEC) under the identifier RBR-10p45rdg (https://ensaiosclinicos.gov.br/rg/RBR-10p45rdg, accessed on 11 June 2024), where the Statistical Analysis Plan (SAP) is available. The Universal Trial Number (UTN) is U1111-1313-4499.

This protocol was developed in accordance with the SPIRIT 2025 Statement. The completed SPIRIT 2025 checklist is provided as Supplementary Material S1.2.

This study is a single randomized controlled trial with blinded outcome assessment, blinded biomarker analyses, and blinded statistical analysis.

The trial has a 2 × 2 factorial structure, defined by two intervention arms BATF vs. Brief Focal Psychotherapy) and two clinical conditions (anxiety vs. depression), resulting in four analytical cells. Participants will be randomized in a 1:1 allocation to intervention arms, with stratification by clinical condition.

The trial includes two pre-specified analytical phases within the same randomized dataset. Phase 1 is the primary confirmatory analysis, comparing BATF versus Brief Focal Psychotherapy with clinical conditions collapsed. Phase 2 is a pre-specified secondary factorial analysis, evaluating the effects of intervention, clinical condition, and their interaction within the 2 × 2 framework. Both phases were defined prior to trial initiation and registered prospectively, with no adaptive modifications or interim decision-making based on Phase 1 results. Publication of Phase 1 results will not influence recruitment, eligibility criteria, randomization procedures, outcome measures, sample size targets, or statistical methods for Phase 2, which will continue according to the prospectively registered protocol.

The design allows estimation of overall treatment efficacy while also enabling pre-specified evaluation of differential effects across clinical conditions within a single randomized controlled trial framework.

### 2.2. Study Timeline

The study will be conducted as a single randomized controlled trial with two pre-planned analytical phases applied to the same underlying dataset.

Phase 1 will include 156 participants and will evaluate the primary outcome comparing BATF and Brief Focal Psychotherapy with collapsed clinical conditions (anxiety and depression). This represents the primary confirmatory analysis of the trial.

Phase 2 will include the full sample (*n* = 472) and will perform a pre-specified 2 × 2 factorial analysis including intervention and clinical condition factors, incorporating all participants from Phase 1. Both analytical phases are pre-specified and independent, with no post hoc modifications to the analytical structure.

Recruitment is anticipated to begin in the fourth quarter of 2026. Recruitment will occur continuously throughout the study period until the target sample size for Phase 2 is reached. Recruitment for Phase 1 is expected to be completed by the fourth quarter of 2027. Intervention delivery and outcome assessments for Phase 1 are expected to be completed shortly thereafter. Recruitment for the full sample is expected to continue until completion of Phase 2, anticipated in the fourth quarter of 2029. Final data collection and database closure are expected following completion of participant follow-up.

### 2.3. Eligibility Criteria

#### 2.3.1. Type of Participants

Students at the Federal University of Rio Grande do Norte (UFRN) seeking outpatient treatment at the mental health unit, in the Psychiatry Division of the Department of Internal Medicine.

#### 2.3.2. Inclusion Criteria

Adults aged 18 years or older with a clinical diagnosis of anxiety and/or depressive disorders will be included in the study upon signing an informed consent form. The diagnosis will be established through a clinical evaluation conducted by trained clinical personnel according to DSM-5-TR diagnostic criteria prior to enrollment. If psychotropic medication is used, it must be used regularly, with no changes in medication type or dose during the 6 weeks preceding study entry. Information regarding medication type, dose, and duration of use will be recorded at baseline. Any medication changes occurring during the study will be documented and considered in sensitivity analyses when appropriate.

#### 2.3.3. Exclusion Criteria

Individuals who meet the following conditions will be excluded from the study: current substance use disorder (excluding alcohol and tobacco use disorders), tobacco use, alcohol use disorder, imminent risk of suicide, schizophrenia, psychotic symptoms, bipolar disorder, use of oral contraceptives, or pregnancy.

Eligibility criteria related to substance use, pregnancy status, medication use, and psychiatric comorbidities will be assessed through clinical interview, self-report, review of available medical information, and, when applicable, confirmation during the baseline clinical evaluation conducted by trained clinical personnel.

These criteria were adopted to minimize potential confounding factors that could influence clinical outcomes and the assessment of salivary and blood biomarkers, ensuring greater homogeneity of the sample and improving the internal validity of the trial.

Participants identified as being at imminent risk of suicide during baseline clinical assessment will not be enrolled and will be immediately referred for appropriate psychiatric care within the Mental Health Unit, including urgent psychiatric evaluation and follow-up when necessary.

### 2.4. Interventions

BATF, the experimental intervention in the present trial, will be delivered in a maximum of three sessions. BATF is an experimental psychotherapeutic intervention currently under evaluation in a randomized controlled trial, and has not yet been validated as an evidence-based treatment. Brief Focal Psychotherapy, which will serve as the active comparator group, may be conducted over a maximum of ten sessions, with sessions occurring once per week.

It is important to note that the difference in the number of sessions between BATF and Brief Focal Psychotherapy reflects the intrinsic structure and clinical logic of each intervention, and not an attempt to equalize treatment dose. BATF was designed as a highly structured, protocol-based brief intervention delivered in a maximum of three sessions, whereas Brief Focal Psychotherapy follows a more conventional brief psychotherapy format, typically delivered over several sessions. Therefore, the present trial compares two distinct models of brief psychotherapy as implemented in clinical practice, rather than interventions with pre-standardized or equivalent dose. This design allows for the evaluation of comparative effectiveness under real-world clinical conditions.

BATF will be implemented on the first day, followed by a second session after four weeks, and a third optional session nine weeks later, if all items from the previous session have not been completed. A follow-up psychotherapy session will be scheduled at week thirteen post-baseline for feedback and the collection of scales and clinical assessments. Session number and treatment duration were defined according to the theoretical and procedural characteristics of each intervention. The timing of BATF sessions was aligned with the predefined assessment schedule of the trial (baseline, 4 weeks, and 13 weeks), which includes clinical scales and biological sample collection (blood and saliva). The second BATF session at 4 weeks coincides with the first post-baseline assessment. The optional third session, scheduled nine weeks after the second session (corresponding to the week-13 follow-up visit), may be conducted when previously established therapeutic objectives have not yet been fully achieved. The 13-week study period was defined to allow completion of Brief Focal Psychotherapy, which may include up to ten sessions, while maintaining a common outcome assessment window for both study arms. This scheduling ensures temporal correspondence between psychotherapeutic intervention, clinical assessments, and biomarker evaluation.

Both the experimental psychotherapy and the standard psychotherapy will be conducted by qualified professionals. These professionals are trained to handle the entire psychotherapeutic process, from diagnosis and therapeutic guidance to clinical assessment of progress.

**Figure 1:**
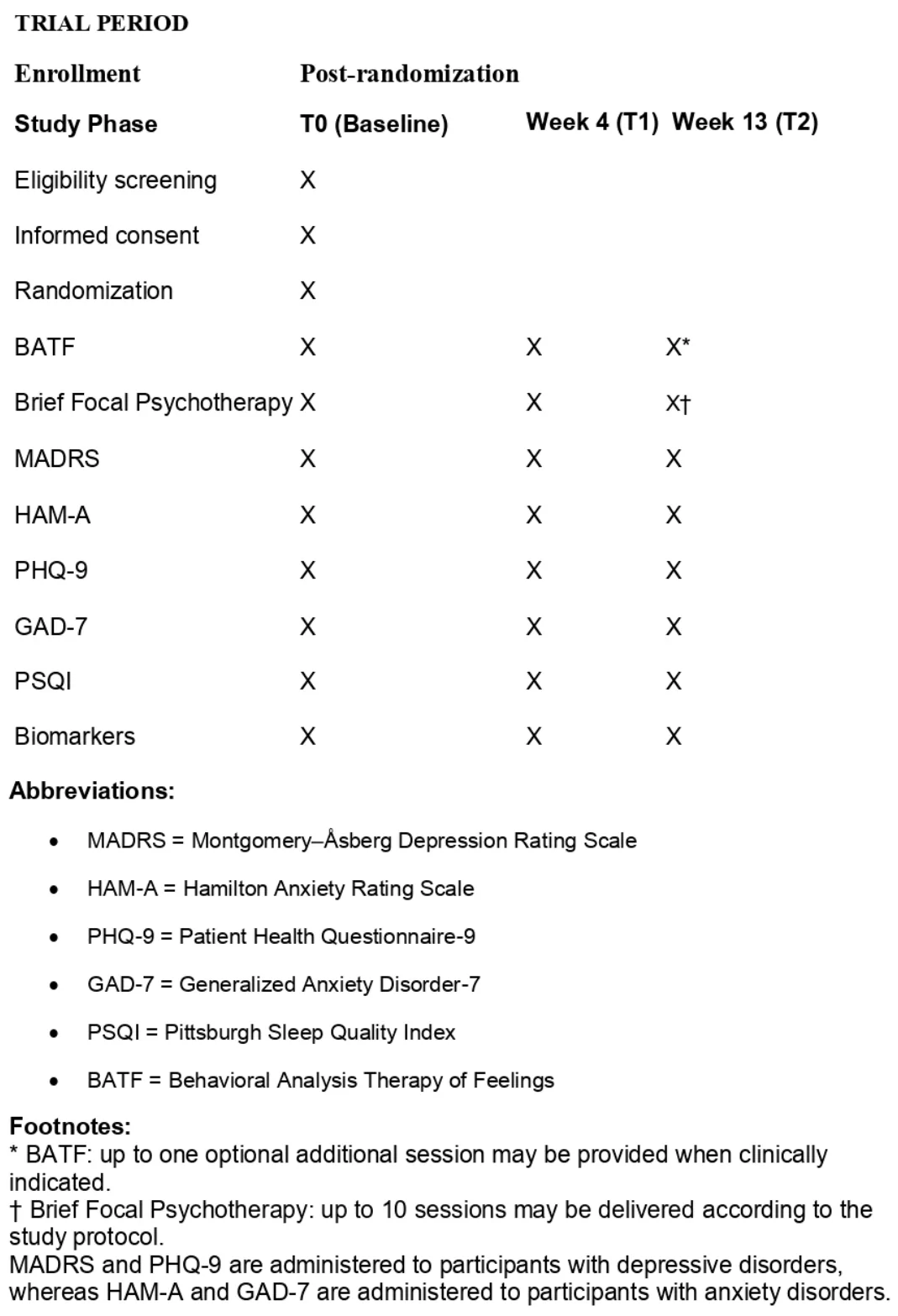
SPIRIT Participant timeline: Schedule of enrollment, interventions, and assessment.

The severity and clinical progression of anxiety and depression will be assessed using well-established and validated clinical scales, together with biomarker analyses of blood and saliva samples. These biomarkers have been extensively investigated in the scientific literature regarding normal physiological ranges and alterations associated with anxiety and depressive symptoms. Therefore, it is important to monitor the levels of each biomarker evolutionarily, from diagnosis through to the end of the patient’s follow-up. Similarly, specific questionnaires for depression and anxiety are established scales for assessing symptom severity from diagnosis, as well as for tracking clinical improvement. The first collection of saliva and blood samples, as well as the administration of depression and anxiety scales, will be conducted prior to the start of psychotherapy to serve as baseline measurements. Subsequent assessments and sample collections will be conducted initially at 4 weeks and again at 13 weeks (Figure 1). Clinical scales and biomarker collections will be synchronized at each assessment time point to ensure temporal correspondence between clinical and biological measures.

Potential Harms, Clinical Deterioration, and Withdrawal Procedures:

As approved by the ethics committee and explained in the informed consent form, all research involving human participants carries some inherent risks, which will be minimized through continuous clinical monitoring during psychotherapy sessions and follow-up assessments.

Adverse Events (AEs):

Adverse events will include any unfavorable psychological, emotional, or clinical occurrence reported by the participant or observed by the therapist during the study period, regardless of direct attribution to the intervention. These may include transient emotional distress, increased anxiety, mood worsening, or discomfort during psychotherapeutic procedures.

Serious Adverse Events (SAEs):

Serious adverse events will include any clinically significant condition requiring urgent psychiatric intervention, hospitalization, emergency referral, substantial functional impairment, suicidal risk requiring immediate evaluation, or any event considered by the investigators to represent significant clinical risk.

Clinical Deterioration:

Clinical deterioration will refer to worsening of anxiety, depressive symptoms, emotional instability, or psychosocial functioning identified through clinical evaluation, psychotherapy sessions, or validated clinical scales during follow-up. If deterioration is suspected, participants may undergo additional diagnostic evaluation using the Structured Clinical Interview for DSM-5-TR (SCID-5-TR) and other clinically relevant assessments.

Clinical Management:

Should symptoms arise requiring pharmacological treatment or additional psychiatric support, psychiatric consultation will be available within the Mental Health Unit of the same institution. Each case will be individually evaluated, and appropriate clinical measures may include extension of psychotherapy, referral to long-term psychotherapy, psychiatric treatment, or other clinically indicated interventions.

Withdrawal Criteria:

Participants may be withdrawn from the study in cases of severe clinical deterioration, serious adverse events, emergence of psychiatric conditions requiring immediate alternative treatment, participant request, non-adherence to study procedures, or investigator judgment that continuation may compromise participant safety or study integrity.

Protocol Deviations:

All protocol deviations, including missed sessions, incomplete assessments, deviations from intervention procedures, or unplanned clinical management modifications, will be documented and reviewed by the investigators. Any clinically relevant deviation affecting participant safety or trial conduct will be reported to the ethics committee when required.

### 2.5. Outcomes

The objective of this study is to evaluate the effects of a brief psychotherapeutic intervention, BATF, compared with Brief Focal Psychotherapy in reducing symptoms of anxiety and depression.

Outcomes include quantitative measures of depressive and anxiety symptoms, sleep quality, and exploratory salivary and blood biomarkers associated with stress, inflammatory processes, and neurobiological function.

The co-primary outcomes for Phase 1 are defined as the change from baseline (Week 0) to Week 13 in symptom severity within each diagnostic stratum, assessed using disorder-specific scales (MADRS for depressive disorder and HAM-A for anxiety disorder).

Clinical diagnoses will be established during baseline psychiatric and clinical evaluation according to DSM-5-TR criteria prior to randomization. Participants will be stratified according to their primary clinical condition (anxiety or depressive disorder) and randomized to one of the two psychotherapeutic interventions. Each participant will be assessed only with the symptom severity scale corresponding to their primary diagnosis.

Secondary outcomes include depressive and anxiety symptom severity assessed using disorder-specific instruments according to participants’ primary clinical condition (PHQ-9 for depressive disorders and GAD-7 for anxiety disorders). Sleep quality, assessed using the Pittsburgh Sleep Quality Index (PSQI), and exploratory biomarker analyses will be performed in all participants.

Exploratory outcomes include salivary and blood biomarkers. Salivary biomarkers include cortisol, glutamate, immunoglobulin A (IgA), lysozyme, melatonin, alpha-amylase, chromogranin A, and fibroblast growth factor 2 (FGF2). Blood biomarkers include cortisol, pro–brain-derived neurotrophic factor (pro-BDNF), mature BDNF, sex hormones, dehydroepiandrosterone (DHEA), C-reactive protein (CRP), growth hormone, and glutamate. Cortisol and glutamate will be assessed in both saliva and blood samples. All biomarkers are pre-specified in the Statistical Analysis Plan and will be considered exploratory and hypothesis-generating.

Assessments will be conducted at baseline (Week 0), Week 4, and Week 13. The primary endpoint is defined as change from baseline (Week 0) to Week 13, and all outcomes will be analyzed according to the pre-specified statistical analysis plan using change-from-baseline metrics. Continuous outcomes will be summarized using group means and standard deviations or medians and interquartile ranges, depending on data distribution.

### 2.6. Recruitment

Participants will access the Mental Health Unit through university-based mental health services, including referrals from student support services as well as spontaneous presentation to the outpatient mental health service. Self-referred participants will be eligible for screening and will undergo the same eligibility assessment procedures and inclusion/exclusion criteria as all other potential participants.

Recruitment will be conducted through standardized institutional communication channels, including university-wide emails, the Integrated Academic Activities Management System (SIGAA), and social media announcements. Recruitment materials will describe the study as a randomized clinical trial comparing two brief psychotherapeutic interventions for anxiety and depressive symptoms. To preserve the integrity of study procedures, recruitment materials will not disclose allocation procedures or specific study hypotheses. However, participants will be informed during the consent process that the study includes an experimental psychotherapeutic intervention, in accordance with ethical requirements for informed consent.

Interested participants will be invited to contact the research team and will subsequently undergo eligibility assessment according to the predefined inclusion and exclusion criteria. Eligible participants who provide written informed consent will proceed to randomization and study enrollment.

### 2.7. Randomization and Blinding

Randomization will be performed after eligibility confirmation and informed consent. The allocation sequence will be generated by an independent researcher using a computer-based randomization procedure, stratified by clinical condition.

Allocation concealment will be ensured using sequentially numbered, opaque, sealed envelopes prepared by a researcher not involved in recruitment, assessment, or intervention delivery.

The envelope will be opened immediately before the psychotherapy session by the therapist responsible for intervention delivery. A confidential file linking participant identifiers to randomization codes will be maintained by the independent researcher who generated the allocation sequence and stored securely in a password-protected cloud system.

Participants will be informed that two psychotherapeutic approaches are being compared, without disclosure of allocation or study hypotheses, to minimize expectancy bias. Distinct researchers will be responsible for randomization, intervention delivery, outcome assessment, biomarker analysis, and statistical analysis, with no cross-access to allocation data during the trial.

The same procedures will be replicated in Phase 2.

### 2.8. Roles and Confidentiality

The study will be conducted at the Onofre Lopes University Hospital (EBSERH), Federal University of Rio Grande do Norte, using institutional clinical infrastructure without additional costs to participants. As there is no external commercial or financial sponsor, no external party is involved in study design, data collection, analysis, interpretation of data, manuscript preparation, or publication decisions. The investigators retain full scientific independence.

Allocation confidentiality will be managed by a designated investigator with restricted access to the randomization key, which will be securely stored in a password-protected file. This role is strictly administrative and limited to data governance.

Clinical interviews for eligibility confirmation will be conducted by trained clinical personnel according to DSM-5-TR criteria prior to inclusion.

Biological samples will be processed and stored by a dedicated researcher responsible for the biorepository, using coded identifiers stratified by sex to ensure confidentiality and traceability.

Statistical analysis will be performed by an independent data analyst using de-identified datasets, ensuring separation from clinical and intervention procedures.

Participant data will be coded at enrollment using official documentation. All datasets will be stored in encrypted, password-protected systems managed by the responsible researchers, with identification keys retained by the principal investigator to ensure confidentiality and traceability.

In cases of dropout or exclusion, participants will be retained in the intention-to-treat analysis, with identification keys remaining under the custody of the principal investigator, and all procedures will be duly documented, including reasons for exclusion, with missing data handled using appropriate statistical methods.

Strategies to ensure treatment adherence include institutional communication, structured scheduling, and clear guidance provided at informed consent, with emphasis on confidentiality and protocol compliance.

No Data Monitoring Committee will be established, and no interim analyses are planned due to the low-risk outpatient nature of the study. The study will be supervised by the investigators with clearly defined roles across trial activities. The principal investigator will oversee protocol compliance without involvement in intervention delivery or data analysis.

Therapist Training and Fidelity:

All interventions will be conducted by licensed clinical psychologists working within the public mental health outpatient service of the Onofre Lopes University Hospital (EBSERH), Federal University of Rio Grande do Norte. They have prior clinical experience in the treatment of participants with anxiety and depressive disorders in routine mental health care. Prior to study initiation, therapists will undergo standardized training in both BATF and Brief Focal Psychotherapy protocols, including theoretical orientation, manual-based procedural instruction, supervised practice, and calibration exercises to ensure fidelity to intervention procedures. Therapists will be trained in both intervention protocols and will be capable of delivering either treatment according to randomized allocation procedures. Intervention delivery will strictly follow study allocation procedures.

BATF is a fully manualized and standardized protocol and will be delivered according to a detailed treatment manual, with the aim of minimizing performance and therapeutic allegiance bias. Treatment fidelity will be ensured through structured post-session checklists, ongoing clinical supervision, regular calibration meetings, and strict adherence to intervention manuals. The investigator clinical psychologist will be responsible for therapist training, inter-therapist calibration, and supervision of intervention delivery to ensure consistency in the application of procedural components, with systematic documentation of any protocol deviations.

### 2.9. Data Collection

#### 2.9.1. Blood and Saliva Collection Procedures

Blood samples will be collected using sterile equipment by trained technicians. A total of 30 mL of blood will be collected in the morning, with the volunteer in a fasted state. On the same days and weeks, the volunteer will also perform three saliva collections at home, at the following intervals: 0, 30, and 45 min after waking up. The cortisol collection at awakening is standardized and must be performed immediately upon waking up, using a salivette, with subsequent collections at 30 and 45 min after waking. The volunteer will receive the salivette and detailed instructions the day before the collection.

To enhance adherence to the saliva sampling protocol, participants will receive standardized written and verbal instructions, and sampling times will be clearly explained during training. Participants will be instructed to record the exact time of awakening and each saliva collection using a structured collection log (self-report diary). Additionally, they will be provided with labeled collection materials corresponding to each time point (0, 30, and 45 min) to reduce timing errors. Compliance will be checked upon sample return, and any deviations from the prescribed timing will be documented and considered in sensitivity analyses when appropriate.

Standardized pre-analytical procedures will be implemented to minimize variability in biomarker measurements. Samples will be collected in the morning under fasting conditions. Participants will be instructed to avoid physical exercise within 48 h prior to collection and to ensure at least 7 h of sleep on the night preceding sampling. Acute illness at the time of assessment will result in rescheduling of sample collection when necessary. Information on medication use, menstrual cycle phase, and use of oral contraceptives will be recorded and considered in sensitivity analyses when appropriate. Caffeine and food intake will be controlled through fasting conditions prior to sample collection.

All collected material, both blood and saliva, will be stored in a biorepository at the Human Measures Laboratory of the Center for Biosciences at the Federal University of Rio Grande do Norte (UFRN), and will be used exclusively for the measurement of the described molecules.

#### 2.9.2. Structured Clinical Interview for DSM-5-TR

The Structured Clinical Interview for DSM-5-TR (SCID-5-TR) will be utilized for screening participants for anxiety and depressive disorders and for determining eligibility prior to randomization. This tool is crucial for selecting subsequent clinical assessment instruments and for establishing the participant’s primary clinical condition. The interview will be administered by trained clinical personnel, including licensed psychologists and psychiatrists from the Mental Health Unit of the Onofre Lopes University Hospital. Interviewers have experience in the assessment of anxiety and depressive disorders and will receive standardized training regarding study procedures before participant recruitment. The interview has been translated and adapted into Portuguese.

#### 2.9.3. Montgomery-Åsberg Depression Rating Scale (MADRS)

The MADRS demonstrates high inter-rater reliability, provides an estimate of severity, and is superior to the Hamilton Depression Rating Scale (HAM-D) in differentiating between responders and non-responders to antidepressant treatment [14].

#### 2.9.4. Clinical and Self-Report Assessment Scales

The Hamilton Anxiety Scale (HAM-A) is among the most widely utilized scales and serves as a reference for newer scales [15]. Self-report measures include the PHQ-9 (for depression), the GAD-7 (for anxiety), and the Pittsburgh Sleep Quality Index.

The GAD-7, a screening tool and severity indicator, allows participants to report their level of discomfort related to anxiety over the past two weeks. The PHQ-9, also a screening tool, provides insight into the patient’s perspective on depressive symptoms over the past two weeks [16]. The PHQ-9 is also a reliable and valid measure of depression severity [17].

The Pittsburgh Sleep Quality Index, with its Brazilian version showing high internal consistency and moderate reliability, assesses sleep quality [18]. The inclusion of these comprehensive assessment tools and the focus on sleep quality reflect the study’s commitment to thoroughly evaluating both the psychological and physiological impacts of the interventions [19].

Both instruments have validated Brazilian Portuguese versions with established psychometric properties [20,21]. These scales will be used in conjunction with clinician-administered instruments (MADRS and HAM-A) to provide complementary assessment of anxiety, depression, and sleep quality.

#### 2.9.5. Salivary Biomarkers and Blood Biomarkers

This study will evaluate clinically relevant salivary and blood biomarkers associated with stress response, inflammation, and neurobiological regulation in anxiety and depressive symptoms. Salivary biomarkers assessed in this study include cortisol, glutamate, immunoglobulin A, lysozyme, melatonin, alpha-amylase, chromogranin A, and fibroblast growth factor 2, which have been implicated in stress reactivity, immune modulation, and neuroendocrine dysregulation in affective disorders [22,23,24,25,26,27].

Blood biomarkers assessed in this study include cortisol, pro-BDNF, mature BDNF, sex hormones, dehydroepiandrosterone, C-reactive protein, growth hormone, and glutamate, which have been linked to depression severity, stress regulation, and inflammatory and neuroplasticity pathways [28,29,30,31,32,33,34,35]. In the present study, BDNF (pro-BDNF and mature BDNF) will be assessed exclusively in blood samples.

Given the exploratory nature of these analyses, biomarker outcomes will be interpreted within an integrated systems framework encompassing hypothalamic–pituitary–adrenal (HPA) axis activity, immune-inflammatory signaling, and neuroplasticity mechanisms.

### 2.10. Sample Size

The standard deviation for the Montgomery-Åsberg Depression Rating Scale (MADRS) was estimated to be 11 [36]. The minimal clinically significant difference on the Montgomery-Åsberg Depression Rating Scale (MADRS) is considered to be 5 points. The minimal clinically important difference for the MADRS is not universally established and varies according to the estimation method and study population. Published estimates generally range from approximately 3 to 9 points, with values around 3–6 points considered among the most plausible across different methodological approaches. Therefore, a 5-point difference was adopted as a pragmatic mid-range estimate for sample size calculation purposes [37]. Alpha (α): a classical significance level of 5% is used, commonly applied in health research. A statistical power of 80% (β = 0.20) was adopted, as commonly recommended in health research.

The sample size was calculated based on the change in Montgomery–Åsberg Depression Rating Scale (MADRS) scores from baseline to week 13 (primary outcome). A two-sided significance level of 5% (α = 0.05), statistical power of 80% (β = 0.20), a clinically meaningful difference of 5 points, a standard deviation of 11, and a 1:1 allocation ratio were assumed. The calculation was performed using a two-sample *t*-test for independent means implemented in G*Power version 3.1.9.7. This corresponds to a standardized effect size of Cohen’s d = 0.45.

#### 2.10.1. Sample Size Calculation for Initial Phase

To test the effect of BATF compared to the active comparator group, and to ensure sample homogeneity by clinical condition, the comparison of mean differences between the two groups is conducted. With the given parameters, the required sample size is 77 participants per group, totaling 154 participants. To account for the need to evenly distribute participants, 78 participants per group are necessary, resulting in a total of 156 participants.

#### 2.10.2. Sample Size for Second Phase

The second phase of the clinical trial will require a larger sample size. Upon reaching 156 participants, the initial results comparing the two psychotherapies (experimental and active comparator groups) will be published. The research methodology will remain unchanged, but the second phase aims to assess the effect of condition across four distinct groups (BATF for depression, BATF for anxiety, control for depression, and control for anxiety).

An Analysis of Variance (ANOVA) will be employed for this comparison. The sample size calculation for this phase requires a total of 472 participants. Therefore, an additional 316 participants need to be recruited, with 79 participants in each of the four groups. This requirement accounts for the data from the initial 156 participants, which will be included in the evaluations for the second phase to achieve the total sample size of 472 for comparison across the four groups.

The sample size calculation for Phase 2 was based on a 2 × 2 factorial design with four equally sized groups, assuming balanced allocation across conditions (*n* = 118 per cell in the final sample). The calculation maintained the same statistical assumptions as Phase 1. No formal interim adjustments were planned, and the design assumes complete case availability at the final analysis stage. Importantly, no attrition (dropout) rate was included in the sample size calculation, which may result in reduced effective statistical power if loss to follow-up occurs during the trial.

### 2.11. Statistical Methods

The primary analysis (Phase 1) will compare BATF versus Brief Focal Psychotherapy in a combined sample of participants with anxiety and/or depressive symptoms, irrespective of diagnostic category (“collapsed” clinical conditions). This analysis represents the primary confirmatory comparison of the trial.

Exploratory subgroup analyses within Phase 1 are pre-specified and will evaluate treatment effects separately in participants with anxiety and depressive symptoms.

The secondary analysis (Phase 2) will examine a pre-specified 2 × 2 factorial analytical framework (intervention × clinical condition), allowing assessment of main and interaction effects across four groups (BATF-depression, BATF-anxiety, control-depression, and control-anxiety), resulting in four analytical cells for the 2 × 2 factorial analysis.

For Phase 1, the primary comparison between BATF and Brief Focal Psychotherapy will be performed using Student’s *t*-test for normally distributed change scores or the Mann–Whitney U test when normality assumptions are not met. The first phase will include 156 participants.

Phase 2 will include a total sample of 472 participants (including participants from Phase 1 plus additional recruited participants). Change scores will be compared across the four predefined groups (BATF-depression, BATF-anxiety, control-depression, and control-anxiety) using one-way analysis of variance (ANOVA) or the Kruskal–Wallis test, as appropriate.

Assessments performed at Week 4 will be used to describe intermediate clinical evolution and may support exploratory analyses of treatment trajectories.

Effect Size Measure: Omega squared (ω^2^); Statistical Significance Level: α = 0.05.

Multiplicity for the co-primary outcomes will be controlled using the Holm–Bonferroni procedure. Secondary and subgroup analyses will be considered exploratory and interpreted accordingly. Exploratory biomarker analyses will apply false discovery rate (FDR) correction according to the Benjamini–Hochberg method.

Primary Analysis: Recruitment will be continuous, and allocation will follow a pre-generated 1:1 randomization sequence, stratified by clinical condition. Participants will be enrolled until the target sample size of randomized participants is reached. All analyses will follow the intention-to-treat principle. Missing outcome data will be handled primarily using maximum likelihood estimation within mixed-effects models. Multiple imputation may be performed as a sensitivity analysis, when appropriate, to evaluate the robustness of the findings.

#### Additional Analyses (Subgroup and Sensitivity)

As a sensitivity analysis, repeated-measures mixed-effects models may be conducted to evaluate the consistency of results across all assessment time points (baseline, Week 4, and Week 13), accounting for within-participant correlation.

Additional sensitivity analyses may include per-protocol analyses restricted to participants who complete the planned intervention and adjusted models controlling for baseline symptom severity, concomitant medication use, and treatment adherence (e.g., number of sessions attended), when appropriate. Post hoc exploratory analyses may be conducted as hypothesis-generating analyses if unanticipated patterns emerge.

## 3. Ethics and Dissemination

The study was approved by the Research Ethics Committee through the Plataforma Brazil. The process number is CEP: 6.555.637 and the CAAE is 74805923.5.0000.5292, approved on 1 December 2025. Version 6. All participants will provide written informed consent prior to inclusion in the study. Informed consent will be obtained by a trained physician or psychologist from the Psychiatry Department of the Onofre Lopes University Hospital.

Importantly, participation or non-participation in this study will have no impact on students’ academic evaluation, academic status, access to university services, or their relationship with faculty members or university staff. All participation is entirely voluntary, and students may withdraw at any time without any academic, institutional, or personal consequences.

Protocol Amendments: any significant modifications to this protocol, such as changes in eligibility criteria, outcomes, or statistical methods, will be reviewed and decided upon by the principal investigator. All amendments will be submitted to the Research Ethics Committee via the Plataforma Brasil for renewed approval and will be updated in the clinical trial registry (ReBEC). These changes will also be reported in study result reports and any related scientific publications.

Use of collected samples in future research: if the use of collected samples is required for a new research project, a new study protocol will be submitted for review by the Institutional Research Ethics Committee (CEP) and, when applicable, by the National Research Ethics Commission (CONEP). Once the project is approved, participants will be re-contacted and asked to sign a new informed consent form (ICF), explicitly authorizing the use of their previously collected samples (saliva and blood) for the new research.

The dissemination of this study will occur through the publication of an open-access article in a peer-reviewed journal, as well as through presentations at academic conferences and scientific seminars. The clinical trial results will also be reported in the Brazilian Clinical Trials Registry (ReBEC).

## 4. Discussion

The present randomized clinical trial adopts a comparative design, evaluating BATF against Brief Focal Psychotherapy, an established brief psychotherapeutic approach in clinical practice [35]. This design allows the assessment of differential treatment effects between a manualized experimental intervention and an active psychotherapeutic comparator. The findings may contribute to the literature on brief psychotherapeutic interventions by providing preliminary evidence on a manualized structured intervention compared with an established brief psychotherapeutic approach, particularly regarding feasibility and clinical outcomes in anxiety and depressive symptoms.

This study may provide information regarding the feasibility and clinical outcomes of a brief structured psychotherapeutic intervention. Broader implications for mental health service organization will depend on the trial results.

A methodological strength of this study is its randomized controlled design, including allocation concealment and separation of roles across randomization, outcome assessment, and statistical analysis. In addition, the use of a manualized intervention protocol enhances reproducibility and reduces treatment heterogeneity.

A key limitation is the inherent inability to blind psychotherapists to treatment allocation due to the nature of psychotherapeutic interventions.

Although participant blinding is not feasible in psychotherapy trials with different session structures, expectancy effects are minimized by presenting both interventions as time-limited brief psychotherapies delivered within a structured clinical framework, without disclosure of allocation or hypothesized superiority.

We acknowledge that recruitment is restricted to treatment-seeking university students from a single academic institution, which may limit the external validity and generalizability of the findings to other populations and clinical settings. Additional limitations include the single-center design, the university student sample, restrictive exclusion criteria, possible therapist allegiance bias, unequal intervention dose between conditions, absence of participant blinding, and the extensive exploratory biomarker assessments. Importantly, the sample size calculation did not include an adjustment for potential attrition (dropout). This may affect the effective statistical power of the study if loss to follow-up occurs during the trial and should be considered when interpreting the results of Phase 2 analyses. Future studies should investigate the applicability of BATF across different clinical populations beyond anxiety and depressive symptoms. Although its procedural framework is based on affective organization and behavioral translation processes that may theoretically be relevant to other psychological conditions involving maladaptive emotional and behavioral functioning, its broader clinical applicability remains to be established through empirical investigation.

## Data Availability

The original contributions presented in this study are included in the article/Supplementary Material. Further inquiries can be directed to the corresponding author.

## Supplementary Materials

The following supporting information can be downloaded at: https://www.mdpi.com/article/10.3390/medsci14050551/s1, S1.1. Procedural Structure and Therapist Manual for Behavioral Analysis Therapy of Feelings (BATF). S1.2. SPIRIT 2025 Checklist.
