# Supplementary material for: Comparative Effects of Behavioral Analysis Therapy of Feelings and Brief Focal Psychotherapy: Protocol for a Randomized Controlled Assessor-Blinded Clinical Trial
Source: Med Sci (Basel). 2026 Sep 8;14(5):551. doi: 10.3390/medsci14050551 (PMC13609297; doi:10.3390/medsci14050551)
Supplement: Supplementary file 1 [file medsci-14-00551-s001.zip › medsci-4369285-supplementary.pdf]

SUPPLEMENTARY MATERIAL S1

Contents

S1.1 Procedural Structure and Therapist Manual for Behavioral Analysis Therapy of Feelings (BATF)

S1.2 SPIRIT 2025 Checklist

S1.1 Procedural Structure and Therapist Manual for Behavioral Analysis Therapy of Feelings (BATF)

1. Overview of the Intervention

The Behavioral Analysis Therapy of Feelings (BATF) is a brief, standardized intervention protocol designed for application in clinical research settings. The intervention aims to support the identification of maladaptive affective patterns and to facilitate the translation of emotional experiences into adaptive behavioral strategies through guided introspection.

BATF is described at the level of procedural implementation required for replication in randomized controlled trials. This document is intended to ensure intervention standardization, procedural reproducibility, and treatment fidelity. Theoretical and conceptual elaborations of the model are beyond the scope of this manuscript and will be addressed in subsequent publications.

2. Theoretical Rationale and Original Contribution of BATF

Behavioral Analysis Therapy of Feelings (BATF) was developed as a brief psychotherapeutic model that integrates affective organization and behavioral translation within a unified procedural sequence. The model is designed to operationalize emotional experience as an intermediate variable linking subjective affect to actionable behavioral planning.

BATF is based on the assumption that clinically relevant affective experiences may not be immediately accessible through reflective cognitive processing alone. The intervention proposes that psychological distress may be partially maintained by difficulties in identifying, organizing, and behaviorally integrating pre-reflective affective experiences. Rather than initially emphasizing interpretation or cognitive restructuring, BATF employs a structured sequence designed to facilitate direct observation of affective experiences in their immediate manifestation. Through sequential affective identification, thematic organization, and behavioral translation, the intervention seeks to increase the cognitive accessibility and functional integration of affective information.

Although BATF was not developed within any single traditional psychotherapeutic school, its procedural structure was informed by a phenomenologically oriented focus on the description of affective experience as immediately lived (Giorgi et al., 2017). BATF shares certain conceptual features with approaches that emphasize emotion regulation and experiential processing, it was developed as an independent intervention and should not be considered a derivative of any established psychotherapeutic school.

BATF was designed to introduce a procedural framework integrating affective organization and behavioral translation within a unified intervention sequence. Emotional states are not only explored or cognitively reframed but systematically reorganized into adaptive–maladaptive contrasts that serve as direct inputs for behavioral goal formulation. This design aims to reduce the procedural distance between emotional processing and behavioral intervention observed across protocol-guided psychotherapeutic approaches.

Within this framework, affect is operationalized as an informational system rather than a secondary output of cognitive appraisal, allowing emotional clustering and thematic recurrence to function as primary inputs for behavioral conversion. This integrated sequence is intended to increase procedural efficiency while maintaining systematic affective exploration within a brief psychotherapeutic format.

The present intervention will be evaluated within an institutional ethics committee-approved randomized controlled trial protocol. The study is currently in the preparatory phase, prior to participant recruitment.

Importantly, BATF was not developed as an application of any single traditional psychotherapeutic school. Rather, its procedural architecture emerged together with its conceptual rationale, which emphasizes the identification, organization, and behavioral integration of affective experiences. The intervention's distinguishing feature is the structured transformation of affective information into behavioral outputs through a fixed sequential system.

Accordingly, the mechanistic hypothesis underlying BATF should be understood as the conceptual rationale that informed the development of the intervention rather than as a post hoc explanation of an independently developed procedure. The model is intended to be empirically evaluated through the present randomized controlled trial.

3. General Structure

Phase 1 — Behavioral Sequence of Affective Analysis

Objective
Identify, organize, and convert affective states related to the presenting problem into thematic elements for subsequent behavioral planning.

Step 1 — Identification of the presenting problem
The therapist asks the participant to describe, in their own words, the primary problem being presented. This description defines the reference context for the procedural sequence.

Step 2 — Initial affective labeling
The participant identifies the first emotional response associated with the presenting problem. This step constitutes the initial point of the affective sequence.

Step 3 — Recording of the first affective state
The identified affective state is documented using a standardized format (e.g., table or recording sheet). Responses are restricted to the description of the immediate affective state, without interpretation, justification, or cognitive elaboration.
The therapist verifies adherence to the experiential level, ensuring that responses remain descriptive.

Step 4 — Sequential elicitation of affective states
The therapist applies a procedural elicitation method in which each identified affective state serves as a stimulus for the identification of additional associated affective states.
The procedure is repeated iteratively until no additional spontaneous responses are generated or previously reported states begin to recur.

Step 5 — Affective transformation (contrast mapping)
For each negative affective state reported, participants identify an alternative affective state perceived as functional or adaptive.
This step operationalizes the contrast between:

- observed affective state;
- corresponding adaptive affective state.

Step 6 — Cognitive association (reflective labeling)
For each adaptive affective state identified, participants report the first interpretation or thought that spontaneously emerges.
The cognitive associations are recorded descriptively, without causal inference or clinical interpretation.

Step 7 — Thematic consolidation
The cognitive associations are analyzed for identification of recurring semantic patterns or conceptual overlaps.
Semantically similar expressions are grouped into thematic clusters.

Step 8 — Reduction of redundancy and thematic selection
Within each thematic cluster, representative elements are selected based on frequency, clarity, and participant validation.
This step produces a reduced set of central themes representative of the participant's cognitive-affective organization.

Step 9 — Prioritization of functional themes
Participants identify which of the selected themes is perceived as most relevant for organizing or integrating the remaining reported content.
The prioritized theme is considered the central organizing element of the participant's reported data.

Step 10 — Behavioral translation
The prioritized theme is used as the basis for initial behavioral planning.
The participant is guided through the following **protocol-guided question**:
"What can I do in practical terms to address this theme in daily life?"
Responses are operationalized into:

- observable behavioral strategies;
- concrete actions;
- initial implementation plans.

Step 11 — Goal formulation and action planning
The responses obtained are organized into initial behavioral goals collaboratively defined by therapist and participant.
Goals are established in terms that are:

- observable;
- feasible;
- contextually specific.

This step constitutes preparation for the subsequent phase of the behavioral intervention.

Phase 2 — Adaptive Conversion and Behavioral Implementation Sequence
Objective
Convert the cognitive-affective themes identified in Phase 1 into adaptive behavioral responses through a **systematic sequence** of emotional conversion, problem-solving procedures, and implementation planning.

Stage 1 — Adaptive conversion questioning
The therapist initiates a protocol-guided adaptive conversion sequence based on the prioritized theme identified in Phase 1.
Participants are guided through reflective prompts intended to identify adaptive abilities, functional responses, or behavioral alternatives associated with the reported affective states.
Protocol-guided question:
"How can an adaptive response be developed in relation to the prioritized theme identified?"
The therapist records the first identified affective state, followed by sequential elicitation of additional related affective states.

Stage 2 — Conversion toward behavioral exposure
The identified affective states are converted into implementation-oriented behavioral responses.
Protocol-guided questions:
"How can this affective state be transformed into a more adaptive response?"
Subsequently:
"What do I need to do to develop this adaptive state?"
Brief socratic questioning may be used to clarify barriers, doubts, or implementation difficulties.
The procedure is repeated iteratively for the remaining identified affective states.

Stage 3 — Solution-focused emotional exploration
Difficulties related to behavioral implementation are used as the basis for a systematic emotional exploration process oriented toward solution development.
Protocol-guided question:
"What do you feel in relation to this problem?"
After registration and sequential elicitation of related affective states, the therapist conducts questioning directed toward the identification of adaptive abilities relevant to resolving the reported difficulty.
Protocol-guided question:
"Which adaptive ability may help in resolving this problem?"
Responses are operationalized into:

- practical strategies;
- functional behavioral responses;
- contextual implementation plans.

Stage 4 — Recognition and refinement of thematic patterns
The responses obtained are analyzed to identify recurring behavioral patterns, implementation barriers, and adaptive strategies.
The identified patterns are collaboratively reviewed and refined when necessary.

Stage 5 — Systematic problem-solving procedures
The prioritized patterns are converted into structured targets for behavioral resolution.
The therapist conducts questioning directed toward the generation of functional solutions and analysis of implementation-related difficulties.
When inconclusive or uncertain responses are produced, the therapist uses reflective questioning to facilitate self-generated clarification and behavioral reorganization.
The following procedures may be used:

- guided reflective questioning;
- socratic dialogue;
- review of previous responses;
- reorganization of previously reported content.

Stage 6 — Therapist-guided behavioral clarification
When necessary, the therapist provides standardized educational clarification or behavioral guidance directed toward implementation planning.
The intervention remains focused on:

- behavioral organization;
- adaptive functioning;
- self-regulation;
- practical implementation strategies.

Non-protocolized interpretative interventions are avoided.

Stage 7 — Behavioral consolidation and between-session exercises
Adaptive responses and behavioral plans are consolidated into:

- observable goals;
- feasible implementation steps;
- protocol-guided behavioral exercises delivered to participants in the form of a structured BATF checklist.

Participants may receive:

- self-monitoring exercises;
- behavioral exposure tasks;
- reflective exercises;
- implementation-oriented activities.

Adherence to the checklist and implementation experiences are reviewed in subsequent sessions.

4. Therapist Training and Standardization

All therapists delivering BATF undergo standardized training prior to participant enrollment. Training includes:

- theoretical orientation to the intervention protocol;
- step-by-step procedural instruction;
- supervised practice sessions;
- role-play simulations;
- calibration of intervention delivery across therapists.

Only therapists who complete all training requirements and demonstrate procedural adherence are authorized to deliver the intervention.

5. Treatment Fidelity Procedures

Treatment fidelity is monitored using standardized procedures.
After each session, therapists complete a standardized checklist documenting:

- completion of mandatory procedural steps;
- adherence to session structure;
- documentation of protocol deviations, if any.

A subset of sessions is independently reviewed during supervision meetings to assess consistency of delivery.
Fidelity is defined as adherence to core procedural components. Minor variations in wording during clinical interaction are permitted, provided that all essential steps are maintained.

6. Between-Session Activities

Participants may be assigned between-session tasks, including:

- reflection on identified emotional themes;
- brief self-monitoring exercises;
- implementation of agreed behavioral actions.

Adherence is reviewed in subsequent sessions based on participant self-report and clinical discussion.

7. Materials Availability

A full therapist manual is available from the corresponding author upon reasonable request.
This Supplementary Material provides a condensed procedural description intended to ensure replicability in clinical trial contexts.
Expanded theoretical and conceptual analyses of BATF will be presented in future publications following completion of the clinical trial.

S1.2 SPIRIT 2025 Checklist

SPIRIT 2025 checklist of items to address in a randomized trial protocol*

Section / Topic	No	SPIRIT 2025 checklist item description	Reported on page no.
**Administrative information**			
Title and structured summary	1a	Title stating the trial design, population, and interventions, with identification as a protocol	**Title page / title.**
	1b	Structured summary of trial design and methods, including items from the World Health Organization Trial Registration Data Set	**Abstract; Ethics and**

			**dissemination section.**
Protocol version	2	Version date and identifier	**Ethics and dissemination section.**
Roles and responsibilities	3a	Names, affiliations, and roles of protocol contributors	**Author Contributions.**
	3b	Name and contact information for the trial sponsor	**Roles and Confidentiality.**
	3c	Role of trial sponsor and funders in design, conduct, analysis, and reporting of trial; including any authority over these activities	**Roles and Confidentiality.**
	3d	Composition, roles, and responsibilities of the coordinating site, steering committee, endpoint adjudication committee, data management team, and other individuals or groups overseeing the trial, if applicable	**Roles and Confidentiality.**
**Open science**			
Trial registration	4	Name of trial registry, identifying number (with URL), and date of registration. If not yet registered, name of intended registry	**Trial Design and Registration.**
Protocol and statistical analysis plan	5	Where the trial protocol and statistical analysis plan can be accessed	**Trial Design and Registration.**
Data sharing	6	Where and how the individual de-identified participant data (including data dictionary), statistical code, and any other materials will be accessible	**Data Availability Statement.**
Funding and conflicts of interest	7a	Sources of funding and other support (e.g., supply of drugs)	**This research received no external funding.; Roles and Confidentiality.**
	7b	Financial and other conflicts of interest for principal investigators and steering committee members	**Competing Interests.**

Dissemination policy	8	Plans to communicate trial results to participants, healthcare professionals, the public, and other relevant groups (e.g., reporting in trial registry, plain language summary, publication)	**Ethics and dissemination section.**
**Introduction**			
Background and rationale	9a	Scientific background and rationale, including summary of relevant studies (published and unpublished) examining benefits and harms for each intervention	**Introdution**
	9b	Explanation for choice of comparator	**Introdution**
Objectives	10	Specific objectives related to benefits and harms	**Introdution; Interventions; Outcome.**
**Methods: Patient and public involvement, trial design**			
Patient and public involvement	11	Details of, or plans for, patient or public involvement in the design, conduct, and reporting of the trial	**N/A**
Trial design	12	Description of trial design including type of trial (e.g., parallel group, crossover), allocation ratio, and framework (e.g., superiority, equivalence, non-inferiority, exploratory)	**Trial Design and Registration; Randomisation and Blinding.**
**Methods: Participants, interventions, and outcomes**			
Trial setting	13	Settings (e.g., community, hospital) and locations (e.g., countries, sites) where the trial will be conducted	**Methods and analysis: Eligibility criteria: Participants; Roles and Confidentiality.**
Eligibility criteria	14a	Eligibility criteria for participants	**Methods and analysis: Eligibility criteria: Participants.**
	14b	If applicable, eligibility criteria for sites and for individuals who will deliver the interventions (e.g., surgeons, physiotherapists)	**Not applicable, as no specific eligibility criteria are required for**

			**intervention providers beyond standard clinical qualification and training.**
Intervention and comparator	15a	Intervention and comparator with sufficient details to allow replication including how, when, and by whom they will be administered. If relevant, where additional materials describing the intervention and comparator (e.g., intervention manual) can be accessed	**Methods and analysis: Interventions.**
	15b	Criteria for discontinuing or modifying allocated intervention/comparator for a trial participant (e.g., drug dose change in response to harms, participant request, or improving/worsening disease)	**Interventions; Roles and Confidentiality.**
	15c	Strategies to improve adherence to intervention/comparator protocols, if applicable, and any procedures for monitoring adherence (e.g., drug tablet return, sessions attended)	**Adherence will be monitored through session attendance records and therapist logs.**
	15d	Concomitant care that is permitted or prohibited during the trial	**Concomitant pharmacological treatment is permitted if stable at baseline. Any changes in medication or initiation of additional psychological treatments during the trial will be documented.**
Outcomes	16	Primary and secondary outcomes, including the specific measurement variable (e.g., systolic blood pressure), analysis metric (e.g., change from baseline, final value, time to event), method of aggregation (e.g., median, proportion), and time point for each outcome	**Outcomes.**
Harms	17	How harms are defined and will be assessed (e.g., systematically, non-systematically)	**Interventions.**
Participant timeline	18	Time schedule of enrollment, interventions (including any run-ins and washouts), assessments, and visits for participants. A schematic diagram is highly recommended (see Figure)	**Figure 1.**

Sample size	19	How sample size was determined, including all assumptions supporting the sample size calculation	**Methods and analysis: Sample size.**
Recruitment	20	Strategies for achieving adequate participant enrollment to reach target sample size	**Methods and analysis: Recruitment.**
**Methods: Assignment of interventions**			
Randomization:			
Sequence generation	21a	Who will generate the random allocation sequence and the method used	**Methods and analysis: Randomisation and Blinding.**
	21b	Type of randomization (simple or restricted) and details of any factors for stratification. To reduce predictability of a random sequence, other details of any planned restriction (e.g., blocking) should be provided in a separate document that is unavailable to those who enroll participants or assign interventions	**Methods and analysis: Randomisation and Blinding; Roles and Confidentiality.**
Allocation concealment mechanism	22	Mechanism used to implement the random allocation sequence (e.g., central computer/telephone; sequentially numbered, opaque, sealed containers), describing any steps to conceal the sequence until interventions are assigned	**Methods and analysis: Randomisation and Blinding.**
Implementation	23	Whether the personnel who will enroll and those who will assign participants to the interventions will have access to the random allocation sequence	**Methods and analysis: Roles and Confidentiality.**
Blinding	24a	Who will be blinded after assignment to interventions (e.g., participants, care providers, outcome assessors, data analysts)	**Methods and analysis: Roles and Confidentiality.**

	24b	If blinded, how blinding will be achieved and description of the similarity of interventions	**Methods and analysis: Roles and Confidentiality.**
	24c	If blinded, circumstances under which unblinding is permissible, and procedure for revealing a participant's allocated intervention during the trial	**Methods and analysis: Roles and Confidentiality.**
**Methods: Data collection, management, and analysis**			
Data collection methods	25a	Plans for assessment and collection of trial data, including any related processes to promote data quality (e.g., duplicate measurements, training of assessors) and a description of trial instruments (e.g., questionnaires, laboratory tests) along with their reliability and validity, if known. Reference to where data collection forms can be accessed, if not in the protocol	**Methods and analysis: Data collection.**
	25b	Plans to promote participant retention and complete follow-up, including list of any outcome data to be collected for participants who discontinue or deviate from intervention protocols	**Methods and analysis: Roles and Confidentiality.**
Data management	26	Plans for data entry, coding, security, and storage, including any related processes to promote data quality (e.g., double data entry; range checks for data values). Reference to where details of data management procedures can be accessed, if not in the protocol	**Methods and analysis: Roles and Confidentiality.**
Statistical methods	27a	Statistical methods used to compare groups for primary and secondary outcomes, including harms	**Methods and analysis: Statistical methods.**
	27b	Definition of who will be included in each analysis (e.g., all randomized participants), and in which group	**Methods and analysis: Statistical methods.**

	27c	How missing data will be handled in the analysis	**Methods and analysis: Statistical methods.**
	27d	Methods for any additional analyses (e.g., subgroup and sensitivity analyses)	**Methods and analysis: Statistical methods.**
**Methods: Monitoring**			
Data monitoring committee	28a	Composition of data monitoring committee (DMC); summary of its role and reporting structure; statement of whether it is independent from the sponsor and funder; conflicts of interest and reference to where further details about its charter can be found, if not in the protocol. Alternatively, an explanation of why a DMC is not needed	**Methods and analysis: Roles and Confidentiality.**
	28b	Explanation of any interim analyses and stopping guidelines, including who will have access to these interim results and make the final decision to terminate the trial	**Methods and analysis: Roles and Confidentiality.**
Trial monitoring	29	Frequency and procedures for monitoring trial conduct. If there is no monitoring, give explanation	**Methods and analysis: Roles and Confidentiality.**
**Ethics**			
Research ethics approval	30	Plans for seeking research ethics committee/institutional review board approval	**Ethics and dissemination section.**
Protocol amendments	31	Plans for communicating important protocol modifications to relevant parties	**Ethics and dissemination section.**
Consent or assent	32a	Who will obtain informed consent or assent from potential trial participants or authorized proxies, and how	**Ethics and dissemination section.**

	32b	Additional consent provisions for collection and use of participant data and biological specimens in ancillary studies, if applicable	**Ethics and dissemination section.**
Confidentiality	33	How personal information about potential and enrolled participants will be collected, shared, and maintained in order to protect confidentiality before, during, and after the trial	**Methods and analysis: Roles and Confidentiality.**
Ancillary and post-trial care	34	Provisions, if any, for ancillary and post-trial care, and for compensation to those who suffer harm from trial participation	**Provided within clinical care structure of the study site; participants will receive appropriate clinical referral if needed.**
